# Cost-Containment of Non-Formulary Medicines Accessibility: Malaysian Experience

**DOI:** 10.1186/2052-3211-8-S1-P14

**Published:** 2015-10-05

**Authors:** Nur Liyana Zainal Bahrin, Yahaya Hassan, Abu Bakar Abd Majeed, Nur Wahida Zulkifli, Azlan Ahmad

**Affiliations:** 1 Department of Pharmacy Practice, Faculty of Pharmacy, University Technology MARA, Bandar Puncak Alam, 42300, Malaysia

## Background

As in Malaysia we are operating the public healthcare system through a fully subsidised moddue to the increasing trends of pharmaceutical expenditures as the Malaysian population grows, the total financial allocation for the MOH has also expanded from RM 15 million in 2010 to RM 16 million in 2012.

## Methods

This study was designed to assess and review current practice and policy in the Malaysia pharmaceutical procurement procedures of non-formulary medicines. This was a quantitative study and was conducted in a cross-sectional study design. A purposive sampling method was applied in the study sample selection. The targeted samples to be involved in the selection were the public healthcare institutions in Malaysia. The study was conducted in the 13 major public hospitals in Malaysia. The data and information on the pharmaceutical procurement of non-formulary medicine management, financial allocation and expenditures on pharmaceuticals purchased in the selected facilities for the three consecutive years of 2011, 2012 and 2013 were collected and analysed statistically. The study has been conducted over a year. The study was carried out from February 2014 until February 2015. The study has been carried out in the major public hospitals in Malaysia focusing both on the out-patient and in-patient sectors.

## Results

The need for the patient healthcare demands are under the purview of the government as we are conducting a subsidised healthcare system. Thus, the availability of medicines in the public institutions is restricted to their formulary. However, in certain circumstances, patient conditions might lead to the need for non-formulary medicines. Thus, this will indirectly lead to the burden of expenditures. In this study, *p* values of 0.046 and 0.007 were obtained for the correlation of the non-formulary medicine cost and the hospital financial allocation. The significant correlation shown has proven that these variables are among the important components in assessing the pharmacy service competency.

## Conclusions

From the study, the definite expenditures of the non-formulary medicines procured were identified. The monetary incurred, facilities involved and quantity of non-formulary medicines procured will be helpful in terms of forecasting pharmaceutical allocation. The outcome obtained aimed to fulfil the research gap in medicine policy towards the financial sustainability of the pharmaceutical services in Malaysian public healthcare institutions.

